# Prognostic Significance of Serum Sodium in Patients With Acute Pulmonary Embolism

**DOI:** 10.14740/jocmr6664

**Published:** 2026-09-26

**Authors:** Patchara Kochaiyapatana, Sukrisd Koowattanatianchai, Jayanton Patumanond, Arwarit Pocathikorn, Sopida Thammongkolchai

**Affiliations:** a Division of Cardiovascular Medicine, Department of Medicine, Faculty of Medicine, Burapha University, Chonburi, Thailand; b Burapha University Hospital, Faculty of Medicine, Burapha University, Chonburi, Thailand; c Center for Clinical Epidemiology and Clinical Statistics, Chiang Mai University Faculty of Medicine, Chiang Mai, Thailand; d Clinical Epidemiology and Medical Statistics Unit, Naresuan University Faculty of Medicine, Tha Pho, Thailand

**Keywords:** Acute pulmonary embolism, Hyponatremia, Serum sodium, Mortality, Prognosis, Survival analysis

## Abstract

**Background:**

Hyponatremia has been associated with adverse outcomes in several cardiovascular diseases; however, its independent prognostic value in patients with acute pulmonary embolism (APE) remains uncertain.

**Methods:**

We conducted a single-center retrospective observational study of 105 hospitalized patients with confirmed APE between January 2020 and June 2025. Baseline clinical characteristics were compared between 1-year survivors and non-survivors. Serum sodium levels at the time of APE diagnosis were assessed, and hyponatremia was defined as a serum sodium level < 135 mmol/L. Univariable and multivariable Cox proportional hazards regression analyses were performed to evaluate the association of serum sodium and other potential prognostic factors with 1-year mortality.

**Results:**

Among 105 hospitalized patients with confirmed APE, 41 (39.0%) died within 1 year. Non-survivors had a higher prevalence of active cancer (61.0% vs. 15.6%, P < 0.001) and chronic obstructive pulmonary disease (COPD) (21.9% vs. 1.6%, P = 0.001), lower systolic blood pressure (108.9 ± 25.9 vs. 130.8 ± 21.6 mm Hg, P<0.001), and higher Pulmonary Embolism Severity Index (PESI) scores (157.3 ± 56.0 vs. 91.0 ± 32.7, P < 0.001) than survivors. Serum sodium at diagnosis was significantly lower in non-survivors than in survivors (130.6 ± 5.9 vs. 136.4 ± 4.4 mmol/L, P < 0.001), and hyponatremia was more frequent among non-survivors (78.1% vs. 35.9%, P < 0.001). In univariable Cox regression, hyponatremia was associated with increased 1-year mortality (univariable hazard ratio (uHR), 4.20; 95% confidence interval (CI), 2.00–8.81; P < 0.001). After adjustment for disease severity and relevant clinical risk factors, serum sodium at diagnosis remained independently associated with 1-year mortality (multivariable hazard ratio (mHR) per 1-mmol/L increase, 0.92; 95% CI, 0.86–0.99; P = 0.043).

**Conclusions:**

Lower serum sodium at diagnosis was independently associated with increased 1-year mortality in hospitalized patients with APE. Hyponatremia may represent a simple and readily available prognostic marker for early risk stratification in APE. Larger prospective studies are needed to confirm these findings.

## Introduction

Acute pulmonary embolism (APE) is the third leading cause of cardiovascular mortality after myocardial infarction and stroke, with an estimated annual incidence of 39–115 cases per 100,000 population and a 30-day mortality rate ranging from 7% to 32% [1–4]. To improve clinical risk assessment and guide management decisions, several prognostic models have been developed, most notably the Pulmonary Embolism Severity Index (PESI) and its simplified version (sPESI). These tools incorporate clinical variables such as age, vital signs, comorbidities, and oxygen saturation to estimate 30-day mortality risk [1, 5]. However, they do not account for laboratory biomarkers, which may provide additional prognostic information and enhance the precision of risk stratification.

Hyponatremia—defined as a serum sodium concentration below 135 mmol/L—is a well-recognized predictor of poor outcomes in various cardiovascular conditions, including heart failure, acute myocardial infarction, pulmonary arterial hypertension, and right ventricular dysfunction [6–8]. The pathophysiology involves neurohormonal activation—including increased levels of vasopressin, norepinephrine, renin, and angiotensin II—resulting in impaired water excretion and dilutional hyponatremia [9, 10]. In left-sided heart failure, for instance, hyponatremia reflects advanced disease and correlates with worse clinical outcomes, including increased hospitalization and mortality rates.

Given the hemodynamic stress and neurohormonal dysregulation seen in APE—especially in cases with right ventricular dysfunction—hyponatremia may similarly serve as a marker of disease severity. Several small-scale studies have suggested an association between admission hyponatremia and poorer outcomes in pulmonary embolism (PE), such as higher mortality and longer hospital stays [3, 4]. Meta-analyses have further shown that hyponatremia is associated with increased in-hospital and 30-day mortality [11, 12]. However, most available evidence has focused primarily on short-term outcomes, while data regarding the independent prognostic value of serum sodium for longer-term mortality remain limited. Therefore, this study aimed to evaluate whether serum sodium at the time of APE diagnosis is independently associated with 1-year mortality in a cohort of hospitalized patients with APE.

## Materials and Methods

### Study design and patients

A retrospective observational cohort study was conducted at Burapha University Hospital, Chonburi, Thailand, involving hospitalized patients diagnosed with APE between January 1, 2020, and June 30, 2025. Burapha University Hospital is a regional, internationally accredited teaching institution, comparable in capability to tertiary care centers. It maintains high clinical standards, qualified personnel, and standardized treatment protocols under national healthcare quality accreditation.

Eligible patients were identified through electronic medical records using the International Classification of Diseases, 10th Revision (ICD-10) codes I26.0 (PE with acute cor pulmonale) and I26.9 (PE without mention of acute cor pulmonale). Inclusion criteria were: (1) age ≥ 18 years; (2) confirmed diagnosis of APE based on at least one of the following: (a) computed tomography pulmonary angiography (CTPA), (b) transthoracic echocardiography findings consistent with APE as defined by the European Society of Cardiology (ESC) guidelines [1], or (c) ventilation-perfusion (V/Q) lung scanning; and (3) documented serum sodium measurements either during hospitalization or within the early postdiagnostic period. Patients were excluded if they had isolated subsegmental PE, chronic thromboembolic pulmonary hypertension, or were lost to follow-up within 30 days of diagnosis. Patients referred to another hospital within this period, for whom subsequent follow-up data were unavailable, were considered lost to follow-up. The study protocol was approved by the Burapha University Institutional Review Board in accordance with the Declaration of Helsinki (Approval No. IRB1-019/2567).

### Clinical data collection

All relevant clinical and laboratory data—including demographics, comorbidities, initial vital signs and hemodynamic parameters, PESI scores and risk classifications, serum sodium levels at the time of APE diagnosis, and high-sensitivity cardiac troponin T (hs-cTnT)—were extracted from the hospital’s electronic medical record system by AP and PK using a standardized data collection form. Diagnoses and imaging findings were cross-checked against the hospital radiology information system, while laboratory results were verified using the laboratory information system. Serum sodium at the time of APE diagnosis was defined as the serum sodium measurement obtained during the initial diagnostic evaluation and early management of the APE episode. Because of the retrospective study design, the exact timing of blood sampling relative to confirmation of APE was not prospectively standardized. Statistical analyses were performed by JP and PK, and data visualization was performed by JP. Because of the retrospective nature of the study, data collection and analysis were not performed under blinded conditions.

Hyponatremia was defined using the conventional threshold of serum sodium < 135 mmol/L. Patients were stratified into two groups based on serum sodium levels at the time of APE diagnosis (hyponatremia group: sodium < 135 mmol/L and normonatremia group: sodium ≥ 135 mmol/L). The institutional laboratory reference range for serum sodium was 135–145 mmol/L. The primary outcome was all-cause mortality within 1 year after the diagnosis of APE. Among patients included in the final cohort, vital status at 1 year after APE diagnosis was successfully ascertained for all patients. Patients who died within 1 year were classified as non-survivors, whereas those who remained alive at 1 year were classified as survivors.

### Statistical analysis

Continuous variables were summarized as mean ± standard deviation (SD), or median with interquartile range (IQR), depending on data distribution. Categorical variables were presented as frequencies and percentages. Comparisons between survivors and non-survivors were conducted using the Mann–Whitney U test for continuous variables and Fisher’s exact test for categorical variables. Univariable and multivariable Cox proportional hazards regression analyses were performed to evaluate the association between clinical variables and mortality outcomes. Variables with a P value < 0.25 in univariable analysis were considered candidate variables for inclusion in the multivariable Cox proportional hazards regression model, using a relatively liberal screening threshold to reduce the likelihood of prematurely excluding potentially relevant covariates [13]. Results were reported as univariable hazard ratios (uHRs) or multivariable hazard ratios (mHRs) with corresponding 95% confidence intervals (CIs). A two-tailed P value < 0.05 was considered statistically significant. All statistical analyses were performed using Stata version 19.0 BE (StataCorp LLC, College Station, TX, USA).

## Results

### Baseline characteristics

A total of 172 patients were initially identified using the prespecified ICD-10 codes I26.0 and I26.9 during the study period. Of these, 136 patients had a confirmed diagnosis of PE, including 132 diagnosed by CTPA and four diagnosed by echocardiography. Thirty-one patients were subsequently excluded: nine with chronic thromboembolic pulmonary hypertension, 18 with isolated subsegmental PE, and four who were lost to follow-up within 30 days after referral to another hospital. The final study cohort therefore comprised 105 patients, all of whom had available 1-year vital status, with no loss to follow-up between 30 days and 1 year (Fig. 1). Among the 105 hospitalized patients with confirmed APE, 41 (39.0%) died within 1 year and 64 survived. Age and sex distribution did not differ significantly between non-survivors and survivors (age: 66.9 ± 12.8 vs. 61.6 ± 20.6 years, P = 0.143; male sex: 41.5% vs. 31.3%, P = 0.303). Non-survivors had significantly lower body mass index (BMI) (23.1 ± 5.1 vs. 25.8 ± 6.0 kg/m^2^, P = 0.020) and body weight (58.9 ± 14.8 vs. 67.8 ± 20.7 kg, P = 0.020). Active cancer was substantially more common among non-survivors than survivors (61.0% vs. 15.6%, P < 0.001), as was chronic obstructive pulmonary disease (COPD) (21.9% vs. 1.6%, P = 0.001). In contrast, diabetes (24.4% vs. 29.7%, P = 0.657), heart failure (12.2% vs. 14.1%, P = 1.000), hyperlipidemia (61.0% vs. 51.6%, P = 0.422), cerebrovascular disease (14.6% vs. 9.4%, P = 0.532), coronary artery disease (4.9% vs. 6.3%, P = 1.000), and previous venous thromboembolism (4.9% vs. 4.7%, P = 1.000) were not significantly different between groups. Recent surgery and trauma were documented in 13 (12.4%) and seven (6.7%) patients, respectively, with no significant differences between non-survivors and survivors (Table 1).

**Figure 1:**
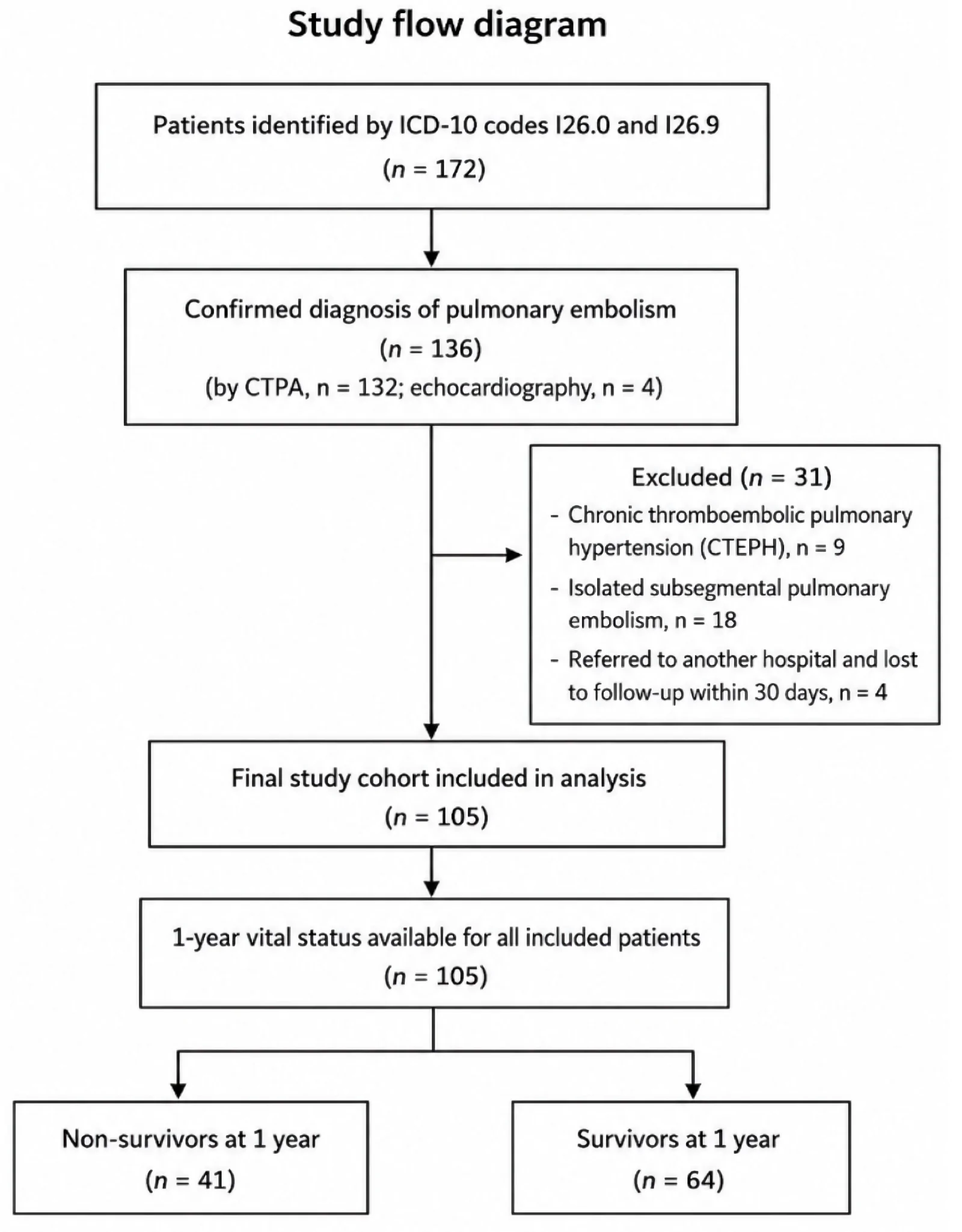
Study flow diagram of patient selection and follow-up. ICD-10: International Classification of Diseases, 10th Revision; CTPA: computed tomography pulmonary angiography.

**Table 1:** Patient Characteristics

Characteristics	Non-survivors (1 year) (n=41)	Survivors (1 year) (n=64)	P value
Male, n (%)	17 (41.5%)	20 (31.3%)	0.303
Age (year), mean ± SD	66.9 ± 12.8	61.6 ± 20.6	0.143
BMI (kg/m^2^), mean ± SD	23.1 ± 5.1	25.8 ± 6.0	0.020
BW (kg), mean ± SD	58.9 ± 14.8	67.8 ± 20.7	0.020
Active cancer, n (%)	25 (61.0%)	10 (15.6%)	< 0.001
Comorbidity, n (%)			
Diabetes	10 (24.4%)	19 (29.7%)	0.657
Heart failure	5 (12.2%)	9 (14.1%)	1.000
Hyperlipidemia	25 (61.0%)	33 (51.6%)	0.422
Cerebrovascular disease	6 (14.6%)	6 (9.4%)	0.532
Coronary artery disease	2 (4.9%)	4 (6.3%)	1.000
Previous VTE	2 (4.9%)	3 (4.7%)	1.000
COPD	9 (21.9%)	1 (1.6%)	0.001
Recent surgery/trauma status, n (%)			
Trauma	1 (2.4%)	6 (9.4%)	0.162
Recent surgery	3 (7.3%)	10 (15.6%)	0.242
Hemodynamic data, mean ± SD			
Heart rate	109.1 ± 20.5	99.9 ± 20.3	0.026
SBP	108.9 ± 25.9	130.8 ± 21.6	< 0.001
DBP	70.2 ± 15.5	79.3 ± 14.6	0.003
MAP	83.11 ± 17.8	96.5 ± 15.0	< 0.001
PESI score, mean ± SD	157.3 ± 56.0	91.0 ± 32.7	< 0.001
PESI classification, n (%)			
Class I	0	10 (15.6%)	< 0.001
Class II	3 (7.3%)	14 (21.9%)	
Class III	6 (14.6%)	24 (37.5%)	
Class IV	5 (12.2%)	11 (17.2%)	
Class V	27 (65.9%)	5 (7.8%)	
Serum sodium at APE diagnosis^a^, mean ± SD	130.6 ± 5.9	136.4 ± 4.4	< 0.001
Hyponatremia (Na < 135), n (%)	32 (78.1%)	23 (35.9%)	< 0.001
Serum troponin, median (IQR) (n = 27,50)	66.6 (36.2–89.5)	41.2 (12.0–81.9)	0.946
Severity of APE, n (%)			
Low	1 (2.4%)	7 (10.9%)	< 0.001
Intermediate-low	10 (24.4%)	23 (35.9%)	
Intermediate-high	14 (34.2%)	32 (50.0%)	
High	16 (39.0%)	2 (3.2%)	
LOS (day), median (IQR)	12.0 (6–21)	12.0 (8–19)	0.659
Follow-up time (day, median) (IQR)	25.0 (10–63)	785.5 (475–1,363)	< 0.001

^a^The institutional reference range for serum sodium was 135–145 mmol/L. APE: acute pulmonary embolism; BMI: body mass index; BW: body weight; COPD: chronic obstructive pulmonary disease; DBP: diastolic blood pressure; IQR: interquartile range; LOS: length of stay; MAP: mean arterial pressure; PESI: Pulmonary Embolism Severity Index; SBP: systolic blood pressure; VTE: venous thromboembolism.

Non-survivors demonstrated more adverse hemodynamic profiles, including higher heart rate (109.1 ± 20.5 vs. 99.9 ± 20.3 beats/min, P = 0.026), lower systolic blood pressure (108.9 ± 25.9 vs. 130.8 ± 21.6 mm Hg, P < 0.001), and lower mean arterial pressure (83.1 ± 17.8 vs. 96.5 ± 15.0 mm Hg, P < 0.001). They also had markedly higher PESI scores than survivors (157.3 ± 56.0 vs. 91.0 ± 32.7, P < 0.001). The overall distribution of PESI classification also differed significantly between groups (P < 0.001), with a greater proportion of non-survivors classified as PESI class V (65.9% vs. 7.8%). Similarly, the overall distribution of APE severity differed significantly between groups (P < 0.001), with high-risk APE occurring more frequently among non-survivors (39.0% vs. 3.2%) (Table 1).

Serum sodium at diagnosis was significantly lower in non-survivors than in survivors (130.6 ± 5.9 vs. 136.4 ± 4.4 mmol/L, P < 0.001). Accordingly, hyponatremia was significantly more common among patients who died within 1 year (78.1% vs. 35.9%, P < 0.001). Serum troponin levels did not differ significantly between non-survivors and survivors (median (IQR), 66.6 (36.2–89.5) vs. 41.2 (12.0–81.9), P = 0.946). Length of hospital stay was also comparable between groups (median (IQR), 12.0 (6–21) vs. 12.0 (8–19) days, P = 0.659) (Table 1).

### Univariable predictors of 1-year mortality

In univariable Cox proportional hazards regression analysis, active cancer (uHR, 4.38; 95% CI, 2.32–8.27; P < 0.001) and COPD (uHR, 3.80; 95% CI, 1.78–8.11; P = 0.001) were associated with increased 1-year mortality. Hemodynamic parameters were also associated with mortality; a higher heart rate was associated with an increased hazard of death (uHR per 1-beat/min increase, 1.02; 95% CI, 1.00–1.03; P = 0.021), whereas higher systolic blood pressure (uHR per 1-mm Hg increase, 0.96; 95% CI, 0.95–0.97; P < 0.001), diastolic blood pressure (uHR, 0.96; 95% CI, 0.94–0.98; P = 0.001), and mean arterial pressure (uHR, 0.96; 95% CI, 0.94–0.97; P < 0.001) were associated with lower hazard (Table 2).

**Table 2:** Univariable Hazard Ratio (uHR) for 1-Year Mortality in Acute Pulmonary Embolism

Prognostic factors	uHR	95% CI	P value
Male	1.39	0.75, 2.60	0.294
Age (year)	1.01	0.99, 1.03	0.216
BMI (kg/m^2^)	0.93	0.87, 0.98	0.017
BW (kg)	0.98	0.96, 0.99	0.024
Active cancer	4.38	2.32, 8.27	< 0.001
Comorbidity			
Heart failure	0.87	0.34, 2.21	0.762
Diabetes	0.82	0.40, 1.68	0.597
Cerebrovascular disease	1.45	0.61, 3.44	0.404
Coronary artery disease	0.90	0.22, 3.72	0.881
Previous VTE	1.08	0.26, 4.48	0.913
COPD	3.80	1.78, 8.11	0.001
Hemodynamic data			
Heart rate	1.02	1.00, 1.03	0.021
SBP	0.96	0.95, 0.97	< 0.001
DBP	0.96	0.94, 0.98	0.001
MAP	0.96	0.94, 0.97	< 0.001
PESI score	1.01	1.01, 1.02	< 0.001
PESI classification^a^	Non-estimable		
Serum sodium at APE diagnosis (mmol/L)	0.86	0.81, 0.91	< 0.001
Hyponatremia (Na < 135 mmol/L)	4.20	2.00, 8.81	< 0.001
Serum troponin	1.0	0.99, 1.0	0.969
Severity of APE			
Low	Reference	—	—
Intermediate-low	2.50	0.32, 19.53	0.383
Intermediate-high	2.54	0.33, 19.37	0.367
High	13.53	1.79, 102.48	0.012

^a^Non-estimable due to zero n in reference category. APE: acute pulmonary embolism; BMI: body mass index; BW: body weight; COPD: chronic obstructive pulmonary disease; DBP: diastolic blood pressure; MAP: mean arterial pressure; PESI: Pulmonary Embolism Severity Index; SBP: systolic blood pressure; VTE: venous thromboembolism; CI: confidence interval; uHR: univariable hazard ratio.

The PESI score was significantly associated with mortality (uHR per 1-point increase, 1.01; 95% CI, 1.01–1.02; P < 0.001). Compared with low-risk APE, high-risk APE was associated with a markedly higher hazard of death, although the CI was wide (uHR, 13.53; 95% CI, 1.79–102.48; P = 0.012). Serum sodium at diagnosis was inversely associated with 1-year mortality (uHR per 1-mmol/L increase, 0.86; 95% CI, 0.81–0.91; P < 0.001), while hyponatremia was associated with more than a fourfold higher hazard of death (uHR, 4.20; 95% CI, 2.00–8.81; P < 0.001). Serum troponin was not significantly associated with mortality (Table 2).

### Multivariable predictors of 1-year mortality

Variables with a P value < 0.25 in univariable analysis were considered for inclusion in the multivariable Cox proportional hazards regression model. PESI classification was excluded because it was directly derived from the continuous PESI score. BMI was also excluded because of its strong correlation with body weight (Pearson’s r = 0.89, P < 0.001); body weight was retained in the multivariable model. In the multivariable model including age, body weight, active cancer, COPD, heart rate, blood pressure parameters, PESI score, and serum sodium at diagnosis, only PESI score and serum sodium remained significantly associated with 1-year mortality.

Each 1-point increase in the PESI score was associated with a higher hazard of death (mHR, 1.01; 95% CI, 1.00–1.02; P = 0.015). Conversely, each 1-mmol/L increase in serum sodium was associated with a lower hazard of death (mHR, 0.92; 95% CI, 0.86–0.99; P = 0.043) (Table 3).

**Table 3:** Multivariable Hazard Ratios (mHRs) for 1-Year Mortality in Acute Pulmonary Embolism

Prognostic factors	mHR	95% CI	P value
Age (per year)	0.98	0.95, 1.01	0.203
BW (per kg)	0.99	0.97, 1.01	0.172
Active cancer	1.73	0.75, 3.95	0.196
COPD	0.75	0.29, 1.98	0.565
Heart rate	0.99	0.98, 1.01	0.596
Systolic blood pressure (per mm Hg)	0.99	0.97, 1.02	0.840
Diastolic blood pressure (per mm Hg)	0.99	0.95, 1.02	0.404
PESI score (per 1-point increase)	1.01	1.00, 1.02	0.015
Serum sodium at APE diagnosis (per mmol/L)	0.92	0.86, 0.99	0.043

The Cox proportional hazards regression model was adjusted for age, body weight, active cancer, chronic obstructive pulmonary disease, heart rate, systolic blood pressure, diastolic blood pressure, Pulmonary Embolism Severity Index score, and serum sodium at APE diagnosis. PESI classification was excluded because it was directly derived from the PESI score. BMI was excluded because of its strong correlation with body weight. APE: acute pulmonary embolism; BW: body weight; COPD: chronic obstructive pulmonary disease; PESI: Pulmonary Embolism Severity Index; CI: confidence interval; mHR: multivariable hazard ratio.

### Independent association of PESI score and serum sodium with mortality hazard

Fractional polynomial plots illustrated the adjusted associations of the PESI score and serum sodium with the estimated mortality hazard. The estimated hazard of death increased progressively with higher PESI scores, with a steeper increase observed at higher score ranges (P = 0.015) (Fig. 2).

**Figure 2:**
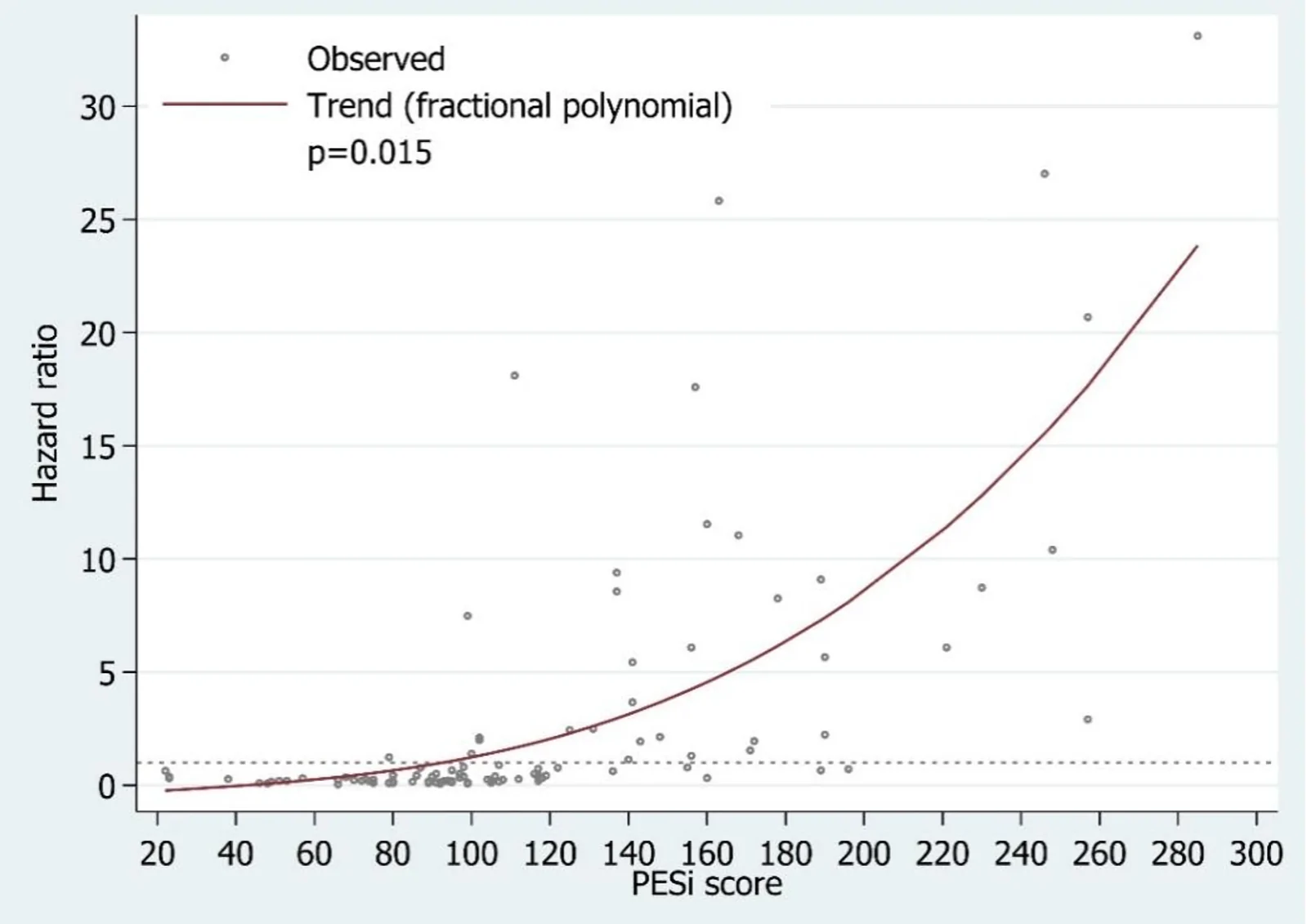
Effect of PESI score on hazard ratio of death within 1 year. PESI: Pulmonary Embolism Severity Index.

For serum sodium, the adjusted hazard of death was higher at lower serum sodium levels and gradually declined as serum sodium increased toward the normal range. An apparent increase in the estimated hazard was observed at higher serum sodium levels, suggesting a possible U-shaped relationship (P = 0.043) (Fig. 3). However, no patients had hypernatremia (> 145 mmol/L), and observations at the upper end of the sodium distribution were limited. When serum sodium was analyzed as a categorical variable, hyponatremia was associated with a higher hazard of death (uHR, 4.20; 95% CI, 2.00–8.81; P < 0.001).

**Figure 3:**
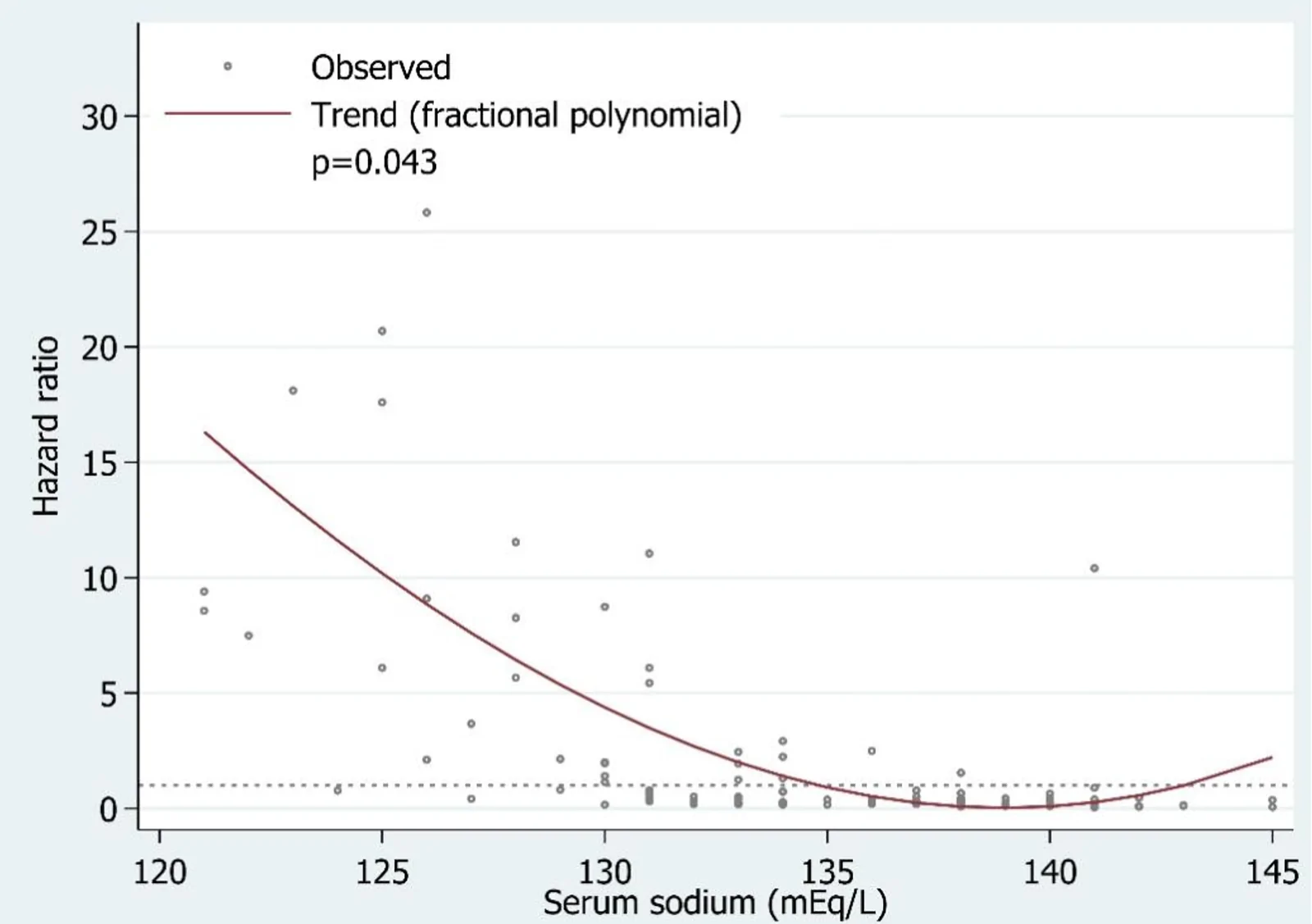
Effect of serum sodium on hazard ratio of death within 1 year.

## Discussion

In this single-center retrospective cohort of hospitalized patients with APE, lower serum sodium at diagnosis was associated with increased 1-year mortality. Non-survivors had more severe clinical profiles, including a higher burden of active cancer and COPD, more adverse hemodynamic parameters, higher PESI scores, and greater APE severity. Hyponatremia was associated with a markedly higher hazard of mortality in univariable analysis, while serum sodium remained independently associated with 1-year mortality after adjustment for disease severity and relevant clinical risk factors.

These findings are clinically relevant given the substantial mortality burden associated with APE. Previous studies have reported 30-day all-cause mortality rates ranging from approximately 7% to 32%, depending on clinical severity and comorbidity burden [3, 4], while longer-term mortality rates, assessed at 1 year or beyond, have ranged from approximately 16% to 38% [4, 14]. In this context, our findings suggest that serum sodium at the time of APE diagnosis may provide additional prognostic information and may serve as a simple adjunctive marker when interpreted alongside established clinical severity parameters.

The association between hyponatremia and adverse outcomes in APE is biologically plausible [15–21]. Hyponatremia in cardiovascular disease is commonly related to neurohormonal activation, including non-osmotic vasopressin release, activation of the renin–angiotensin–aldosterone system, and sympathetic nervous system stimulation. These mechanisms impair free-water clearance and may lead to dilutional hyponatremia, particularly in patients with reduced effective arterial circulating volume or hemodynamic compromise [10, 22]. In the setting of APE, acute right ventricular pressure overload may reduce left ventricular preload and systemic cardiac output. This may trigger neurohormonal responses and water retention, contributing to lower serum sodium levels. In our cohort, non-survivors had higher heart rates, lower systolic and mean arterial pressures, higher PESI scores, and more frequent high-risk APE, suggesting that hyponatremia may partly reflect the severity of cardiopulmonary compromise. However, hyponatremia in APE is unlikely to be explained by hemodynamic factors alone. Non-survivors also had a markedly higher prevalence of active cancer and COPD. These comorbidities may contribute to hyponatremia through multiple mechanisms, including systemic inflammation [23–25], poor oral intake [23], medication use [23], renal dysfunction [23], inappropriate antidiuretic hormone secretion [23, 24, 26], and malignancy-related processes [23, 27]. Therefore, hyponatremia may represent an integrated marker of APE severity, comorbidity burden, and systemic illness rather than a direct causal factor for mortality [16, 28]. Although several relevant comorbidities and biomarkers were evaluated, some were not retained in the multivariable model based on the prespecified variable-selection criteria, and residual confounding cannot therefore be completely excluded.

The fractional polynomial analysis suggested a possible nonlinear relationship between serum sodium and mortality, with an apparent increase in the estimated hazard at the upper end of the normal sodium range. However, no patients had hypernatremia (> 145 mmol/L), and only a small number had serum sodium > 140 mmol/L. Therefore, this apparent upturn should be interpreted cautiously and cannot be considered evidence of an adverse prognostic effect of hypernatremia in APE.

The PESI score remained significantly associated with 1-year mortality, reinforcing its established role in APE risk stratification. Serum sodium at diagnosis also showed an inverse association with mortality and remained significantly associated with 1-year mortality after adjustment for disease severity and relevant clinical risk factors. Because serum sodium is routinely measured, inexpensive, and rapidly available, it may provide additional prognostic information during early assessment of patients with APE. Nevertheless, serum sodium should not be interpreted in isolation or used as a replacement for standard risk stratification tools. Rather, hyponatremia should be considered an adjunctive marker within the broader clinical context, particularly alongside PESI score, hemodynamic status, right ventricular assessment, cardiac biomarkers, and comorbidity burden.

This study also has several limitations. First, this was a retrospective single-center cohort study, which may limit generalizability. Second, the modest sample size and limited number of events may have affected the precision of the multivariable estimates. Although a relatively liberal univariable screening threshold was used for covariate selection [13], some clinically relevant variables may not have been retained in the multivariable model. Therefore, residual confounding from variables not included in the final model cannot be completely excluded. Third, serum sodium was measured only at the time of APE diagnosis, and serial changes were not evaluated. In addition, because the timing of serum sodium measurement relative to intravenous fluid administration was not standardized, the potential influence of fluid-related changes in serum sodium could not be completely excluded. Finally, because the outcome was all-cause mortality, PE-related deaths could not be distinguished from deaths due to comorbid conditions or other causes.

### Conclusions

Serum sodium at the time of APE diagnosis was independently associated with 1-year mortality and may provide simple adjunctive prognostic information during early risk assessment. However, serum sodium should be interpreted within the broader clinical context and not as a replacement for established risk stratification tools, particularly PESI score. Future prospective studies are needed to validate these findings and evaluate whether serum sodium can improve existing prognostic models for APE.

## Data Availability

The data supporting the findings of this study are available from the corresponding author upon reasonable request.

## References

[1] Konstantinides SV, Meyer G, Becattini C, Bueno H, Geersing GJ, Harjola VP, Huisman MV (2020). 2019 ESC Guidelines for the diagnosis and management of acute pulmonary embolism developed in collaboration with the European Respiratory Society (ERS). Eur Heart J.

[2] Wendelboe AM, Raskob GE (2016). Global burden of thrombosis: epidemiologic aspects. Circ Res.

[3] Gerardo F, Faria D, Silverio Antonio P, Baltazar Ferreira J, Beringuilho M, Ferreira H, Fialho I (2024). Prognosis in Pulmonary Embolism (PoPE): 30-Day mortality risk score based on five admission parameters. Rev Port Cardiol.

[4] Madsen SF, Christensen DM, Strange JE, Nouhravesh N, Kumler T, Gislason G, Lamberts M (2023). Short- and long-term mortality for patients with and without a cancer diagnosis following pulmonary embolism in Denmark, 2000 to 2020: A Nationwide Study. J Am Heart Assoc.

[5] Creager MA, Barnes GD, Giri J, Mukherjee D, Jones WS, Burnett AE, Writing Committee Members (2026). 2026 AHA/ACC/ACCP/ACEP/CHEST/SCAI/SHM/SIR/SVM/SVN guideline for the evaluation and management of acute pulmonary embolism in adults: a report of the American College of Cardiology/American Heart Association Joint Committee on Clinical Practice Guidelines. Circulation.

[6] Goldberg A, Hammerman H, Petcherski S, Nassar M, Zdorovyak A, Yalonetsky S, Kapeliovich M (2006). Hyponatremia and long-term mortality in survivors of acute ST-elevation myocardial infarction. Arch Intern Med.

[7] Guerreiro I, Magalhaes H, Coelho S, Ribeiro A, Ferreira S, Araujo E, Bettencourt P (2019). Long term prognosis of acute pulmonary embolism. Eur J Intern Med.

[8] Upadhyay A, Jaber BL, Madias NE (2006). Incidence and prevalence of hyponatremia. Am J Med.

[9] Schrier RW, Abraham WT (1999). Hormones and hemodynamics in heart failure. N Engl J Med.

[10] Verbalis JG (2003). Disorders of body water homeostasis. Best Pract Res Clin Endocrinol Metab.

[11] You W, Fan XY, Chen Y, Wang XL, Song J, Nie CC, Dong Q (2026). Risk factors for mortality in patients with pulmonary embolism-a meta-analysis. J Intensive Care Med.

[12] Zhou XY, Chen HL, Ni SS (2017). Hyponatremia and short-term prognosis of patients with acute pulmonary embolism: A meta-analysis. Int J Cardiol.

[13] Bursac Z, Gauss CH, Williams DK, Hosmer DW (2008). Purposeful selection of variables in logistic regression. Source Code Biol Med.

[14] Ng AC, Chung T, Yong AS, Wong HS, Chow V, Celermajer DS, Kritharides L (2011). Long-term cardiovascular and noncardiovascular mortality of 1023 patients with confirmed acute pulmonary embolism. Circ Cardiovasc Qual Outcomes.

[15] Korkmaz I, Guven FM, Eren SH, Beydilli I, Yildirim B, Aktas C, Alagozlu H (2013). Baseline characteristics and the association between hyponatraemia and pulmonary embolism prognosis. J Pak Med Assoc.

[16] Scherz N, Labarere J, Mean M, Ibrahim SA, Fine MJ, Aujesky D (2010). Prognostic importance of hyponatremia in patients with acute pulmonary embolism. Am J Respir Crit Care Med.

[17] Wong CC, Ng AC, Lau JK, Chow V, Chen V, Ng AC, Yong AS (2016). High mortality in patients presenting with acute pulmonary embolism and elevated INR not on anticoagulant therapy. Thromb Haemost.

[18] Agrawal N, Ramegowda RT, Patra S, Hegde M, Agarwal A, Kolhari V, Gupta K (2014). Predictors of inhospital prognosis in acute pulmonary embolism: keeping it simple and effective!. Blood Coagul Fibrinolysis.

[19] Ng AC, Chow V, Yong AS, Chung T, Kritharides L (2013). Fluctuation of serum sodium and its impact on short and long-term mortality following acute pulmonary embolism. PLoS One.

[20] Ng AC, Chow V, Yong AS, Chung T, Kritharides L (2013). Prognostic impact of the Charlson comorbidity index on mortality following acute pulmonary embolism. Respiration.

[21] Ouatu A, Mihai MS, Tanase DM, Dascalu CG, Dima N, Serban LI, Rezus C (2021). Hyponatremia-long-term prognostic factor for nonfatal pulmonary embolism. Diagnostics (Basel).

[22] Schrier RW (2006). Water and sodium retention in edematous disorders: role of vasopressin and aldosterone. Am J Med.

[23] Adrogue HJ, Tucker BM, Madias NE (2022). Diagnosis and management of hyponatremia: a review. JAMA.

[24] Ayus JC, Negri AL, Moritz ML, Lee KM, Caputo D, Borda ME, Go AS (2021). Hyponatremia, inflammation at admission, and mortality in hospitalized COVID-19 patients: a prospective cohort study. Front Med (Lausanne).

[25] Swart RM, Hoorn EJ, Betjes MG, Zietse R (2011). Hyponatremia and inflammation: the emerging role of interleukin-6 in osmoregulation. Nephron Physiol.

[26] Adrogue HJ, Madias NE (2023). The syndrome of inappropriate antidiuresis. N Engl J Med.

[27] Goldvaser H, Rozen-Zvi B, Yerushalmi R, Gafter-Gvili A, Lahav M, Shepshelovich D (2016). Malignancy associated SIADH: characterization and clinical implications. Acta Oncol.

[28] Tamizifar B, Kheiry S, Fereidoony F (2015). Hyponatremia and 30 days mortality of patients with acute pulmonary embolism. J Res Med Sci.

